# PTSD treatment impacts on pain: secondary analysis of sertraline, prolonged exposure, and their combination

**DOI:** 10.1080/20008066.2026.2727209

**Published:** 2026-09-21

**Authors:** Sheila A.M. Rauch, H. Myra Kim, Natalie Hellman, Ron Acierno, Naomi M. Simon, Anthony P. King, Carolyn B. Allard

**Affiliations:** a Mental Health Service Line, Joseph Maxwell Cleland Atlanta VA Medical Center, Decatur, GA, USA; b Department of Psychiatry and Behavioral Sciences, Emory University School of Medicine, Atlanta, GA, USA; c Office of Vice President for Research, University of Michigan, Ann Arbor, MI, USA; d Prisma Health Upstate, Prisma Health, Greenville, SC, USA; e Department of Family Medicine, University of South Carolina School of Medicine at Greenville, Greenville, SC, USA; f Faillace Department of Psychiatry and Behavioral Sciences, University of Texas Health Science Center at Houston, Houston, TX, USA; g Ralph H. Johnson VA Health Care System, Charleston, SC, USA; h Department of Psychiatry, New York University Grossman School of Medicine, New York, NY, USA; i Department of Psychiatry and Behavioral Health, Institute for Behavioral Medicine, The Ohio State University, Columbus, OH, USA; j Clinical Psychology PhD Program, California School of Professional Psychology, Alliant International University, San Diego, CA, USA

**Keywords:** Pain, PTSD, veterans, treatment, exposure therapy, SSRI, Dolor, TEPT, veteranos, tratamiento, terapia de exposición, ISRS

## Abstract

**Background:** Although posttraumatic stress disorder (PTSD) and pain commonly co-occur, interventions often target them separately. No studies have compared the impact of PTSD psychotherapy, pharmacotherapy, and their combination on pain.

**Objectives:** This secondary analysis of a PTSD effectiveness trial examined the impact of PTSD treatments on pain complaints in 196 veterans randomized to three conditions: Prolonged Exposure plus placebo (PE + PLB), PE + Sertraline (PE + SERT), and SERT with enhanced medication management, overall and among those who reported clinically significant pain at baseline.

**Method:** Linear mixed models assessed change on the Brief Pain Inventory (Pain Severity and Interference subscales) over 52 weeks across treatments in the overall sample, and in those with clinically significant baseline pain interference (≥ 5, *n* = 70) and baseline pain severity (≥ 5, *n* = 93).

**Results:** Veterans overall, and those with clinically significant baseline pain interference and severity, showed significant reductions in pain interference and severity scores across time in all treatments. The PE + PLB group showed greater reductions in pain interference during treatment than SERT and PE + SERT. Veterans with clinically significant baseline pain severity showed significant reduction in pain severity across time in all treatments, with no evidence for differential rates across treatments.

**Conclusion:** While it is uncertain reduced pain was due to the treatments themselves, lower scores for all treatments suggest that PTSD-focused treatments may reduce pain even without a specific pain focus. Research is needed to isolate, if present, mechanistic pathways between PTSD treatment and pain reduction.

**Trial registration:** ClinicalTrials.gov identifier: NCT01524133.

## HIGHLIGHTS

- Pain reduced across PTSD-focused treatments overall, and in those with significant baseline pain.
- Additional research should isolate the mechanisms to determine if the impact of PTSD treatment on pain is causal.
- For some, treating PTSD may provide lasting reduction in pain.

## Abstract (es)

**Antecedentes:** Aunque el trastorno por estrés postraumático (TEPT) y el dolor suelen presentarse de forma conjunta, las intervenciones suelen abordarlos por separado. No hay estudios que hayan comparado el impacto de la psicoterapia, la farmacoterapia y la combinación de ambas en el TEPT sobre el dolor.

**Objetivos:** Este análisis secundario de un ensayo sobre la eficacia del TEPT examinó el impacto de los tratamientos del TEPT en las quejas de dolor en 196 veteranos asignados aleatoriamente a tres grupos: exposición prolongada más placebo (EP + PLB), EP + sertralina (EP + SERT) y SERT con gestión farmacológica optimizada, tanto en general como entre aquellos que referían dolor clínicamente significativo al inicio del estudio.

**Método:** Se utilizaron modelos lineales mixtos para evaluar la evolución en el Inventario Breve del Dolor (subescalas de «Gravedad del dolor» e «Interferencia del dolor») a lo largo de 52 semanas en todos los tratamientos, tanto en la muestra general como en aquellos con una interferencia del dolor clínicamente significativa al inicio del estudio (≥ 5, n = 70) y gravedad del dolor basal (≥ 5, n = 93)

**Resultados:** En general, los veteranos, y aquellos con una interferencia y una gravedad del dolor clínicamente significativas al inicio del estudio, mostraron reducciones significativas en las puntuaciones de interferencia y gravedad del dolor a lo largo del tiempo en todos los tratamientos. El grupo EP + PLB mostró mayores reducciones en la interferencia del dolor durante el tratamiento que los grupos SERT y EP + SERT. Los veteranos con una gravedad del dolor clínicamente significativa al inicio del estudio mostraron una reducción significativa de la gravedad del dolor a lo largo del tiempo en todos los tratamientos, sin que se observaran diferencias en las tasas de reducción entre los distintos tratamientos.

**Conclusión:** Aunque no está claro si la reducción del dolor se debió a los propios tratamientos, las puntuaciones más bajas observadas en todos los tratamientos sugieren que los tratamientos centrados en el TEPT pueden reducir el dolor incluso sin un enfoque específico en el dolor. Es necesario investigar para identificar, si existen, las vías mecánicas entre el tratamiento del TEPT y la reducción del dolor.

## 1. Introduction

Posttraumatic stress disorder (PTSD) is twice as prevalent in veterans relative to the general US population (11–12% versus 6%; Goldberg et al., 2019). Among all war era veterans, 66% of those with PTSD also had a chronic pain diagnosis (Shipherd et al., 2007). Among veterans in a chronic pain program, 49% met PTSD criteria (Otis et al., 2010). As such, there is high co-occurrence. PTSD severity is related to more negative health perceptions including pain (Rauch et al., 2006). Veterans with co-occurring chronic pain and PTSD utilize healthcare services at a higher rate than those with pain or PTSD alone (Benedict et al., 2020). In addition, both PTSD and pain are associated with increased risk for suicide (Magruder et al., 2012) and low rates of treatment completion (Kehle-Forbes et al., 2016). Finally, veterans with both conditions are more likely to be prescribed opioid medications and more likely to incur harmful consequences of these medications (Bernardy & Montano, 2019; Seal et al., 2012). Thus, co-occurrence of PTSD and pain creates a high-risk group that may require additional intervention or modification to current interventions to engage and optimize treatment.

Two theoretical models developed to explain high co-occurrence of chronic pain and PTSD include mutual maintenance theory (Sharp & Harvey, 2001) and shared vulnerability (Asmundson et al., 2002). Shared vulnerability posits that pain and PTSD co-occurrence result from shared biological and psychological risk factors that similarly impact coping post-trauma. Mutual maintenance theory asserts that both pain and PTSD are developed and/or maintained through bi-directional attentional biases, cognitive and behavioural avoidance, and anxiety and pain sensitivity. Mutual maintenance theory hypothesizes that changes in PTSD symptoms precipitate changes in chronic pain, and vice versa (Sharp & Harvey, 2001). While the influence of pain-related cognitions was highlighted in mutual maintenance theory, the role of posttraumatic cognitions was not specifically noted. Recent developments in PTSD treatment research support that changes in negative posttraumatic cognitions are a powerful mechanism of change in PTSD symptoms (Kumpula et al., 2017; Rauch et al., 2015) and in chronic pain (Torres et al., 2024). In PTSD treatment-seeking veteran samples, severity of negative posttraumatic cognitions partially mediates the relationship between PTSD severity and pain interference (Porter et al., 2013), and mediates the relationship between post-concussive symptoms and pain severity and interference (Avallone et al., 2019). Interestingly, another study found that patients who were more active, despite high reported pain severity and interference, had fewer PTSD symptoms (Bourn et al., 2016), possibly due to mutual maintenance, in that as activity increases, more positive interpretations of pain and PTSD symptoms occur. In addition, and consistent with shared vulnerability theory, past research demonstrates that pain and PTSD share specific neuroanatomy and neurobiological underpinnings (Hellman et al., 2025; Scioli-Salter et al., 2015). A recent theoretical and clinical application paper presented a neuroscience-based model for how physical and emotional pain in PTSD may coincide and respond simultaneously to interventions and applied this in a model for integrating chronic pain into Prolonged Exposure (PE) for PTSD consistent with emotional processing theory (Hellman et al., 2025).

Several pain interventions show efficacy, but effect sizes are small to medium overall, and many patients drop out prior to treatment completion (Veterans Affairs (VA)/Department of Defense (DOD), 2022). In addition, some studies show that psychotherapies effective in reducing PTSD symptoms may also reduce pain (Goldstein et al., 2019; Kovacevic et al., 2024). In a recent meta-analysis of the impact of nonpharmacological interventions on pain and PTSD, trauma-focused interventions, such as PE, showed significant improvements in PTSD and pain intensity, leading to a recommendation to include a trauma-focused component when targeting this population (O'Donnell et al., 2026). To our knowledge, no study has examined the comparative impact of PTSD-focused psychotherapy, pharmacotherapy, and their combination on pain.

Additional interventions or modifications to current interventions may be needed to optimize the impact of treatment on both pain and PTSD for this population. For instance, PE and sertraline are effective treatments for PTSD (VA/DOD, 2023), but higher baseline pain predicts poorer treatment response to sertraline and/or PE (Rauch et al., 2021). Several combined interventions are promising. In an open feasibility trial, transcranial direct current stimulation (tDCS) combined with PE showed significant reductions in pain interference and PTSD severity, but not pain severity (Hernandez-Tejada et al., 2023). Also, an integrated protocol of cognitive behavioural therapy for co-occurring pain and PTSD significantly reduced both (Otis et al., 2024). Pain-focused psychoeducation and exercise (PNE) delivered over four weeks showed clinically meaningful reduction in PTSD symptoms and disability at short-term follow-up among those with PTSD and chronic low back pain (Benedict et al., 2024). PNE reduced both pain avoidance and negative pain beliefs and improved PTSD symptoms, suggesting a mutual maintenance model of change. A collaborative care model using (1) behavioural activation (Plagge et al., 2013), (2) an internet-based acceptance and commitment therapy (Åkerblom et al., 2025), and (3) heart rate variability-focused biofeedback (Chadwick et al., 2026) also significantly reduced PTSD symptoms and pain interference. Results of these combined interventions are promising, though effect sizes for pain interference measures were generally small and similar to those of pain and PTSD alone treatments, suggesting room for improvement on pain treatment.

The current study is a secondary analysis examining change in pain complaints during PTSD treatment (Avallone et al., 2019). We focused first on the full PTSD treatment sample to provide an examination of pain response in a broad PTSD treatment sample as a benchmark of change in pain in PTSD-focused clinical trials that do not recruit specifically for pain co-occurrence. This overall analysis may underestimate the impact of PTSD treatment on pain because it includes veterans who are not reporting clinically significant pain at baseline. We therefore also analyzed pain response for veterans with clinically significant baseline pain (operationally defined below). We hypothesized that pain severity and interference would reduce with PTSD treatment in both samples. Since the primary outcome study (Avallone et al., 2019) found no treatment arm differences in PTSD outcomes, we did not hypothesize differential treatment arm effects on pain severity or interference.

## 2. Methods

### 2.1. Participants & procedures

One hundred ninety-six veterans participated in a randomized clinical trial comparing 24 weeks of treatment with PE plus placebo (PE + PLB), PE + Sertraline (PE + SERT), and Sertraline plus enhanced medication management (SERT; Rauch et al., 2019). The current analyses were secondary examinations of pain complaints that include acute and chronic pain as we did not operationalize that the reported pain must be chronic. Readers are referred to (Rauch et al., 2018) for complete study design details. Sites included VA Ann Arbor Healthcare System (VAAAHS), VA San Diego Healthcare System (VASDHS), Ralph H. Johnson VA Medical Center (RHJVAMC), and Massachusetts General Hospital Home Base Veterans Program (MGH). Each site’s Institutional Review Board and the Department of Defense (DOD) Human Research Protection Office (HRPO) approved the PROlonGed ExpoSure and Sertraline Trial (PROGrESS) protocol. The overall study is registered at ClinicalTrials.gov (NCT01524133). All participants provided informed consent prior to enrollment. Participants and providers were blind to pill condition through week 24 (study primary endpoint) and evaluators were blind to treatment assignments. Assessments occurred at baseline (week 0) and weeks 6, 12, 24, 36 and 52. Gender was assessed by veteran report only.

Inclusion criteria were combat-related PTSD and significant impairment (i.e. Clinicians-Administered PTSD Scale [CAPS] ≥ 50) of at least three months' duration. Exclusion criteria were: (1) current, imminent risk of suicide; (2) active psychosis; (3) alcohol or substance dependence (past 8 weeks); (4) inability to attend weekly appointments for the treatment period; (5) prior intolerance or nonresponse trial of PE or SERT; (6) medical illness that contraindicated study treatment; (7) serious cognitive impairment that interfered with study participation (e.g. inability to track discussion); and (8) concurrent antidepressants or antipsychotics, benzodiazepines, prazosin, and sleep agents (e.g. zolpidem) if the dose was not stable for 2 weeks by baseline. A total of 207 veterans were randomized to three treatment conditions and dispensed medication/placebo. Analyses for the current study included the 196 (94.7%) veterans with baseline pain data. PE + PLB arm veterans had somewhat greater baseline CAPS PTSD severity and pain severity (Table 1) at baseline. No patient demographics differed across treatments.

**Table 1.** Baseline characteristics of the study cohort, by treatment conditions and overall (*N* = 196).

	SERT	PE + PLB	PE + SERT	Total	*p*-
	*N* = 67	*N* = 65	*N* = 64	*N* *=* *196*	Value^a^
Age, years	33.3 (8.3)	34.8 (8.4)	34.9 (8.5)	34.3 (8.4)	.31
Male, *n* (%)	62 (92.5%)	58 (89.2%)	52 (81.3%)	172 (87.8%)	.50
Race, *n* (%)					
White	41 (61.2%)	35 (53.9%)	39 (60.9%)	115 (58.7%)	.74
Black	18 (26.9%)	19 (29.2%)	19 (29.7%)	56 (28.6%)	
Other	8 (11.9%)	11 (16.9%)	6 (9.4%)	25 (12.8%)	
Hispanic or Latino Ethnicity, *n* (%)	14 (20.9%)	7 (10.8%)	9 (14.1%)	31 (15.3%)	.09
Marital Status^b^, *n* (%)					
Married	39 (59.1%)	35 (53.9%)	28 (43.8%)	102 (52.3%)	.06
Never married	19 (28.8%)	10 (15.4%)	14 (21.9%)	43 (22.1%)	
Divorced	7 (10.6%)	14 (21.5%)	16 (25.0%)	37 (19.0%)	
Separated	1 (1.5%)	6 (9.2%)	6 (9.4%)	13 (6.7%)	
Education, *n* (%)					
High School (or equivalent)	30 (44.8%)	22 (33.9%)	18 (28.1%)	70 (35.7%)	.16
Some College (13-15 years)	29 (43.3%)	27 (41.5%)	34 (53.1%)	90 (45.9%)	
Bachelor’s or Above (16+)	8 (11.9%)	16 (24.6%)	12 (18.9%)	36 (18.8%)	
Work Status, *n* (%)					
Full Time	33 (49.2%)	32 (49.2%)	33 (51.6%)	98 (50.0%)	.89
Part Time	6 (9.0%)	9 (13.9%)	8 (12.5%)	23 (11.7%)	
Not Working	28 (41.8%)	24 (36.9%)	23 (35.9%)	75 (38.3%)	
Time since trauma^b^, years	7.1 (2.4)	7.3 (3.4)	6.9 (3.1)	7.1 (3.0)	.69
Clinician-administered symptom (CAPS)	75.1 (14.6)	81.0 (13.4)	75.6 (14.3)	77.2 (14.3)	.02
Self-report PTSD symptom (PCL-S)	56.2 (10.2)	59.6 (9.7)	56.3 (11.4)	57.4 (10.5)	.06
Depression (BDI-II)	23.3 (10.2)	26.8 (11.3)	23.4 (10.9)	24.5 (10.9)	.06
Somatic Symptoms (PHQ-15)^b^	12.6 (5.4)	13.1 (4.8)	13.0 (5.0)	12.9 (6.5)	.55
Pain Severity (BPI)	4.1 (2.4)	5.0 (2.1)	4.6 (2.3)	4.6 (2.3)	.01
Pain Interference (BPI)^b^	3.8 (2.7)	4.3 (2.5)	3.9 (2.5)	4.0 (2.6)	.22
Guilt Cognition (TRGI)^b^	0.9 (0.7)	1.1 (0.8)	0.9 (0.6)	1.0 (0.7)	.19
No. of Drinks per Day^b^	2.9 (4.2)	2.5 (3.6)	2.9 (3.3)	2.8 (3.3)	.53

Note: Cell values are mean (standard deviation) unless otherwise noted.

^a^ From comparing across the three treatment arms, stratified by study sites.

^b^ 1 person missing marital status; 8 missing time since trauma; 1 missing PHQ15; 3 missing BPI pain interference; 5 missing TRGI, 3 missing drinking data.

Abbreviations: BDI-II = Beck Depression Inventory-Second Edition; BPI is brief pain inventory; CAPS = Clinician-Administered Post Traumatic Symptom Disorder Scale, total score from 17 items for the past month; PCL-S is PTSD Checklist Specific Stressor Version; PE is prolonged exposure; PLB is placebo; PHQ-15 is 15-symptom Patient Health Questionnaire; SERT is sertraline; TRGI is Trauma Related Guilt Inventory.

### 2.2. Interventions

For detailed treatment descriptions, readers are referred to the primary methodology and outcomes papers (Avallone et al., 2019; Rauch et al., 2018). Active treatment began at week 0 and was maintained through week 24.

PE (Foa et al., 2019) is an effective, first-line treatment for PTSD across clinical practice guidelines (Association, 2025; Forbes et al., 2020; VA/DOD, 2023). PE has three components: (1) psychoeducation about trauma, PTSD, and benefits of approaching trauma memories and reminders, (2) in vivo exposure (i.e. approaching previously avoided but relatively safe trauma-related people, places, and situations), and (3) imaginal exposure and processing (i.e. approaching and revisiting the trauma memory or memories). Participants were scheduled for 13 standard, 90-minute PE sessions by week 12 and were required to complete them by week 24.

Sertraline is a serotonin-specific reuptake inhibitor that is recommended in guidelines for management of chronic pain (NICE, 2021) and PTSD (American Psychological Association, 2025; VA/DOD, 2023). Sertraline was titrated (flexibly adjusted between 50 and 200 mg/day) through week 10 and continued until week 24. Medication management (sertraline or placebo) was fully manualized. Pills were administered alongside PE or enhanced medication management for those in SERT alone to balance time, psychoeducation, and provider support between conditions (Rauch et al., 2018). The SERT condition that did not include PE included clear instructions for providers to not talk about trauma details, include elements of exposure, or give guidance on addressing PTSD-specific symptoms. PE + SERT included all elements of both conditions.

### 2.3. Measures

Brief Pain Inventory (BPI; Cleeland & Ryan, 1994) pain severity and pain interference subscale scores were the primary outcomes. Since no specific duration of pain was required on the BPI, ratings include both new onset and more chronic pain. The 9-item self-report BPI assessed four summative pain severity indices (least, worst, right now, average) and functional interference in seven areas from 0 (no pain/interference) to 10 (worst possible pain/completely interferes) on a scale. BPI has excellent reliability and validity (Cleeland & Ryan, 1994). Pain severity was the mean of four pain intensity items and pain functional interference was the mean of seven functional interference items. Clinically significant baseline pain severity and interference were identified by scores ≥ 5 on each respective subscale, as this indicates moderate pain and as recommended by scale developers (Cleeland & Ryan, 1994).

Clinician-Administered PTSD Scale (CAPS; Blake et al., 1995) is a semi-structured interview assessing PTSD symptom severity in the past month with excellent reliability and validity (Weathers et al., 2001). It was the primary outcome for the original study (Avallone et al., 2019). The DSM-IV version of CAPS was used as the study started prior to DSM-5.

### 2.4. Analytic plan

The study biostatistician (Kim) led the analysis plan and implementation. Primary outcomes were pain severity and pain interference. Apart from adding baseline PTSD severity as a control per reviewer suggestion, the analytic plan was established *a priori*, though not registered. To understand the impact of PTSD treatment on pain in veterans with PTSD, we first obtained summary statistics (means and standard deviations) and visualized means of each pain outcome at baseline (week 0) and weeks 6, 12, 24, 36 and 52, by treatment arms. We used linear mixed models for the pain interference and severity outcomes separately, including all timepoints with change from baseline during the follow-up assessment times as dependent variables to assess if the rate and amount of pain reduction differed by treatment arms (PE + PLB, SERT, and PE + SERT). The model included treatment arm, time (in weeks), time-by-arm interaction terms, baseline values of pain measures, baseline PTSD severity, study site (stratification factor), and random intercepts for participants and random time slopes to allow heterogeneity in rate of change in pain over time across participants. For each pain outcome, a lack of significant time-by-treatment interaction terms based on a likelihood ratio test (LRT) indicated no evidence for differential rates of change in pain across groups and led to refitting the reduced model after dropping the interaction terms. We then tested for the time effect to see if the magnitude of pain change continued to increase or decrease over time, and lack of a significant time effect led to further dropping of time. Refitting with only arm indicators allowed testing if time-averaged pain changes differed across arms. Lastly, when neither time nor arm effects were found, we fit a model adjusting only for baseline values of pain measure, study site, and random intercepts and slopes, which provided an expected magnitude of pain change averaged across all timepoints and arms. Finally, these same analytic steps for each pain outcome were repeated in veterans with clinically significant baseline pain on the specified outcome. Marital status, gender, race, and age were included in the model as covariates. Age is a robust correlate with pain (Mullins et al., 2022). All other covariates were chosen because of baseline differences found across treatment arm (marital status and gender) or association with missingness of outcome data (race) in the main treatment study (Avallone et al., 2019). Cohen’s *d* effect size was used to interpret effects (Cohen, 1992).

Under the assumption of missingness at random, where missingness depends on other observed variables but not the missing values themselves, a mixed model adjusting for baseline variables associated with missingness is expected to provide unbiased estimates (Molenberghs et al., 2008). To include pain data of participants in the model with only baseline pain assessments, we also fit a similar mixed model using raw pain outcome scores from baseline to week 52; the model additionally included a post-baseline time indicator to estimate the pain reduction magnitude during follow-up.

## 3. Results

### 3.1. Overall sample

Cross-sectional means over time reduced for both pain interference and pain severity for the full sample (*N* = 196; Table 2), with no difference in time slopes across treatment arms in pain interference (LRT for interaction effects χ^2^(2) = 5.07, *p* = .08) or severity change (LRT χ^2^(2) = 2.95, *p* = .23) based on a mixed model with time by treatment arm interaction terms. After dropping the interaction terms, we found neither time nor arms were significantly different for pain interference (time effect *p* = .35; arm effect χ^2^(2) = 2.78, *p* = .25) or severity (time effect *p* = .73; arm effect χ^2^(2) = 1.58, *p* = .45), indicating no difference in magnitude of pain change over time or across arms. We therefore dropped both time and treatment arm variables and estimated the magnitude of pain reduction averaged across arms and time, controlling for baseline values of the pain outcome variable, study sites, marital status, age, and race and accounting for random intercepts and slopes. Marginal mean reduction in pain interference from baseline across three groups and follow-up was 0.73 (*p* < .001, 95% CI [0.49, 0.97]), corresponding to a standardized effect size Cohen’s *d* of 0.38 (standardized using model-based total variability). Pain severity also reduced significantly from baseline, with a marginal mean change across three groups at follow-up of 0.58 (*p* < .001; 95% CI [0.37, 0.78], Cohen’s *d* = 0.33).

**Table 2.** Unadjusted mean patient-reported pain at baseline and at post-treatment follow-up times by treatment arm in overall sample of patients with PTSD (*N* = 196).

	Treatment	Time since randomization			
		Baseline	Week 6	Week 24	Week 52
Brief Pain Inventory (BPI) subscales, cell values are mean (SD)					
Pain Interference	SERT	3.8 (2.7)	3.0 (2.6)	3.3 (2.7)	2.8 (3.1)
(possible range: 0–10)		Change^a^	0.8 (2.4)	0.8 (2.2)	1.3 (2.5)
	PE + PLB	4.3 (2.5)	4.0 (2.3)	3.7 (2.5)	3.4 (2.6)
		Change^a^	0.4 (2.1)	0.8 (2.3)	1.1 (1.8)
	PE + SERT	3.9 (2.5)	3.4 (2.9)	3.6 (2.8)	3.9 (2.3)
		Change^a^	0.7 (1.9)	0.4 (2.1)	0.4 (1.8)
Pain severity	SERT	4.1 (2.4)	3.7 (2.5)	4.0 (2.6)	3.6 (3.0)
(possible range: 0-10)		Change^a^	0.5 (1.8)	0.4 (2.4)	0.5 (2.2)
	PE + PLB	5.0 (2.1)	4.7 (2.3)	4.3 (2.3)	4.1 (1.9)
		Change^a^	0.5 (1.5)	1.0 (1.9)	1.1 (1.9)
	PE + SERT	4.6 (2.3)	4.2 (2.5)	4.3 (2.6)	4.8 (2.4)
		Change^a^	0.6 (1.7)	0.4 (2.0)	0.2 (1.9)

Abbreviation: NA is not assessed.

Note: *N* of patients providing the brief pain inventory data were 196, 159, 132, 124 at baseline, week 6, week 24, and week 52, respectively

^a^ Changes from baseline for the two brief pain inventory subscales; larger positive changes imply reduction in pain outcomes for BPI subscales.

Twenty-seven participants had only baseline pain data, and a mixed model fit using raw pain data from baseline to week 52 gave similar estimates regarding magnitude of pain reduction during follow-up for both interference and severity. In the overall sample, clinically significant pain interference (scores ≥ 5) was reported in 36.3% (70/193) at baseline, 31.6% (42/133) at week 24, and 28.2% (35/124) at week 52. Clinically significant pain severity (scores ≥ 5) was reported in 47.4% (93/196) at baseline, 40.2% (53/132) at week 24, and 38.7% (49/124) at week 52.

### 3.2. Veterans with clinically significant baseline pain interference

**Figure 1.**
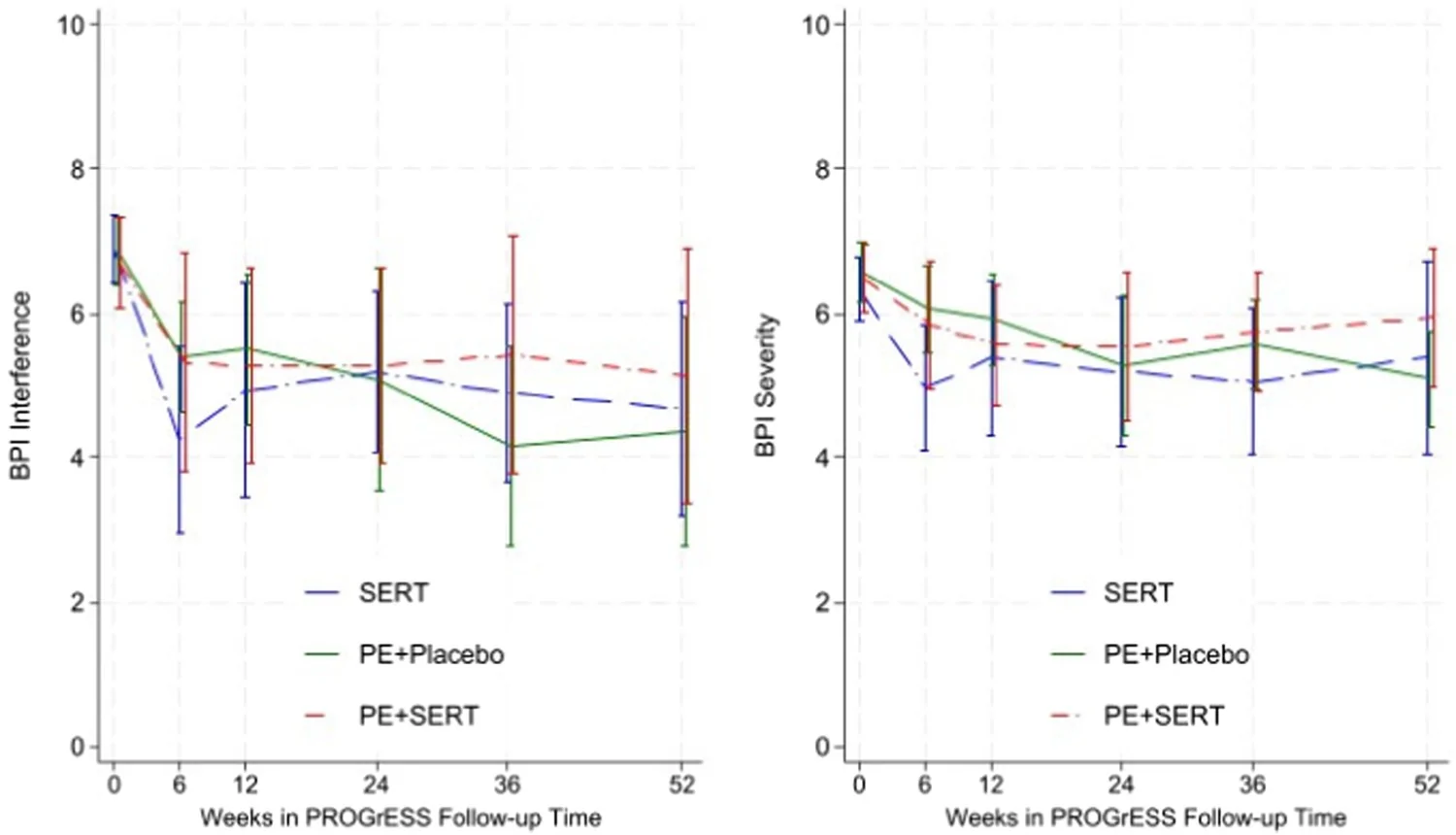
Unadjusted mean pain interference and severity over time by treatment group in those with clinically significant baseline pain interference (≥5) or pain severity (≥5) over 52 weeks.

Of the 196 combat veterans, 70 (35.7%) reported clinically significant pain interference (scores ≥ 5). A covariate-adjusted model showed significantly different rates of change over time in pain interference reduction in this group, where those in the PE + PLB treatment arm showed a different time slope relative to SERT arm veterans (*p* = .03; Table 3, Figure 1). Linear contrasts of coefficients showed pain interference reduction continued to decrease over time in PE + PLB arm veterans at a rate of 0.03 per week (*p* = .02; 95% CI [0.006, 0.051]), while no time effect was seen in pain interference change for veterans in SERT (*p* = .59) and in PE + SERT (*p* = .40). As pain interference reduction continued to improve over time only in PE + PLB arm veterans, we illustrated the treatment arm difference by examining the marginal mean pain interference reduction at the end of follow-up. At one year, the covariate-adjusted predicted mean reduction in pain interference was greatest for veterans in PE + PLB (2.90, *p* < .001; 95% CI [1.84, 3.95]), compared to SERT (1.85, *p* < .001; 95% CI [0.90, 2.80]) and PE + SERT (1.80, *p* = .001, 95% CI [0.74, 2.86]) arms.

**Table 3.** Longitudinal data mixed model of change (baseline minus follow-up times) in pain interference and change in pain severity assessed at weeks 6, 12, 24, 36 and 52 among veterans with PTSD symptoms and clinically significant baseline pain ≥5.

	Beta	Std. Err.	*p*-value	95% CI	
				Lower	Upper
**Pain Interference Change**^a^ **(*n*** **=** **70)**					
Baseline Pain Interference	0.02	0.19	0.94	−0.36	0.39
PE + PLB (Ref: SERT + EMM)	−0.72	0.57	0.20	−1.84	0.39
PE + SERT (Ref: SERT + EMM)	−0.84	0.61	0.17	−2.02	0.35
Time since randomization (weeks)	−0.005	.01	0.59	−0.03	0.01
**Time by PE** **+** **PLB**	**0**.**03**	**0**.**02**	**0**.**03**	**0**.**00**	**0**.**06**
Time by PE + SERT	0.01	0.02	0.32	−0.02	0.04
**Pain Severity Change**^a^^,^^b^ **(*n*** **=** **93)**					
Baseline Pain Severity	0.15	0.13	0.24	−0.10	0.41
PE + PLB (Ref: SERT + EMM)	−0.82	0.46	0.07	−1.72	0.08
PE + SERT (Ref: SERT + EMM)	−0.90	0.49	0.07	−1.86	0.06
Time since randomization (weeks)	−0.01	0.01	0.53	−0.02	0.01
**Time by PE** **+** **PLB**	0.02	0.01	0.10	−0.00	0.04
Time by PE + SERT	0.003	.011	0.78	−0.02	0.03

^a^ Each pain interference change and pain severity change model was fit using a mixed model with changes from baseline at weeks 6, 12, 24, 36 and 52 as dependent variables, adjusting for study sites (stratification factor), marital status, age, sex and race, and included random intercepts and time slopes.

^b^ The model for pain severity change is shown to parallel the model to that of pain interference change. The overall magnitude of pain severity reduction provided under Results section was estimated by a simpler model after dropping one after another time by arm interaction terms, arm indicators, and time, as none of these were statistically significant, but adjusting for baseline values of pain severity, study sites (stratification factor), marital status, age, sex and race, and included random intercepts and time slopes.

Abbreviations: PE is prolonged exposure; PTSD is posttraumatic symptom disorder; SERT is sertraline; PLB is placebo.

### 3.3. Veterans with clinically significant baseline pain severity

Baseline clinically significant pain severity (scores ≥ 5) was reported in 93 (47.4%) of 196 veterans. In these veterans, we did not find a differential time effect across arms on pain severity change (LRT for interaction terms χ^2^[2] = 3.03, *p* = .22, Table 3, Figure 1), although PE + PLB arm veterans showed a marginally significant trend toward continued improvement in pain severity over the follow-up at a rate of 0.01 (*p* = .09) per week. After dropping the time by arm interaction terms, we did not find time slope (*p* = .66) to be significant, and after dropping the time slope, we did not find arm effects to be significant (χ^2^[2] = 4.22, *p* = .12); therefore, we did not find evidence for a difference across arms in the time-averaged magnitudes for the reduction in pain severity. However, the overall change from baseline in pain severity across time and arms in veterans with clinically significant baseline pain was significant with a marginal mean change of 0.98 (*p* < .001, 95% CI [0.68, 1.28], Cohen’s *d* = 0.56), adjusting for baseline pain severity, covariates, and random intercepts and slopes.

## 4. Discussion

As hypothesized, among the full sample of veterans receiving PTSD treatment, overall pain interference and severity were both significantly reduced across all conditions (PE + PLB, PE + SERT, SERT). Indeed, the BPI reduction based on the model was 0.73 points (Cohen’s *d* = 0.38, small) in pain interference and 0.58 points (Cohen’s *d* = 0.33, small) in severity over treatment and follow-up (Cohen, 1992). This full sample included both veterans who did and did not report clinically significant pain at baseline and thus may underestimate the magnitude of effect we would expect for those with both PTSD and significant pain. In addition, because the study did not specify the duration of pain at baseline, some of this reduction may be related to resolution of acute pain and not attributed to the intervention. Thus, the effect of PTSD treatment alone on pain requires additional examination to determine its importance.

Roughly 47% of veterans at baseline and 39% of veterans at week 52 reported clinically significant pain severity. Thus, the co-occurrence was common and PTSD treatment coincided with pain severity reductions evident for one year. Across groups for those with clinically significant pain, the average change in pain severity with PTSD-focused intervention alone was 0.98 points. This is similar to a combined PTSD and chronic pain intervention called Emotional Awareness and Expression Therapy (Lumley et al., 2017; Yarns et al., 2024) that showed a reduction of 1.26 points in pain severity over six months. While this shows reduction in pain, it is unclear if it can be attributed to PTSD treatment, and further reductions would have been preferable (i.e. pain was not resolved), indicating potential relevance of a more focused pain intervention.

Roughly 36% of veterans at baseline and 28% at week 52 reported clinically significant pain interference. Thus, as with pain severity, pain interference reduced overall. Of note, veterans in PE + PLB group showed continued improvement over the follow-up time and larger reductions in pain interference than either the SERT or PE + SERT groups at week 52, suggesting PE affects pain interference more than medication. Indeed, it is possible that veteran attribution of change may lead to greater change overall in PE + PLB than the other arms. Specifically, all veterans were unblinded at week 24 and those who received PE + PLB reported more reduction from that point forward in pain interference than those assigned to either group that received active sertraline. Thus, those given sertraline may have attributed change in pain to the pill and subsequently reduced engagement with an exposure-based lifestyle after that point to a greater degree than those who received placebo and attributed their changes to their own work in the treatment. Additional examination of this pattern of change and whether attribution is contributing to symptom reduction in pain during follow-up is warranted.

As noted in the introduction, change in negative posttraumatic cognitions, including negative thoughts about the self as damaged or broken, are a powerful mechanism of change in PTSD symptoms (Kumpula et al., 2017; Rauch et al., 2015) and are related to pain in PTSD (Avallone et al., 2019; Porter et al., 2013). Reductions in pain in the current study suggest that when someone with PTSD engages with life activities – whether due to treatment with exposure or other reasons – this engagement is associated with improved function related to PTSD and pain reduction. Thus, a bidirectional relationship between PTSD and pain, where positive change in one leads to change in the other, (Asmundson et al., 2002; Sharp & Harvey, 2001) may be especially supported in trauma-focused interventions including exposure-based interventions (O'Donnell et al., 2026).

No studies prior to our analysis have examined the comparative impact of effective psychological, pharmacological, and combined treatments for PTSD alone on pain. Change in PTSD severity and interference found in the current study was similar in magnitude to that shown in other specific pain and combined pain and PTSD interventions (Åkerblom et al., 2025; Lumley et al., 2017; Plagge et al., 2013; Yarns et al., 2024). Consistent with mutual maintenance theory, effectively reducing PTSD with treatment was associated with a significant reduction in pain. PTSD treatment alone, whether PE + PLB, SERT, or PE + SERT, led to reductions in some that were maintained over time. However, many veterans continued to report clinically significant pain (scores ≥ 5) at the end of treatment. Additional studies are needed to determine whether more focus on pain during PTSD episodes of treatment can achieve further reductions in pain interference and severity for those with this co-occurrence, without reducing the magnitude of PTSD treatment response. A combined intervention for PTSD and pain could provide efficient improvement in symptoms and quality of life. Care systems may also benefit through saved provider training and time. Recent work on pain and emotional processing theory (Hellman et al., 2025) provides direction on how to integrate psychoeducation and pain-based in vivo exposures into the current model of PE for PTSD, aiming to grow the impact on pain while not interfering with PTSD treatment response.

### 4.1. Limitations

While the findings from this first comparative study of PTSD psychological and pharmacological therapy on pain are exciting, important limitations warrant discussion. Replication in other veteran samples with co-occurring PTSD and pain is needed. As a secondary analysis of pain that was not powered for examination among those with clinically significant baseline pain, findings should not be over-interpreted. The PE + PLB group had more severe clinical symptoms at baseline, and this may partially explain some of the differences found. While we controlled for these differences, replication in other veteran samples with co-occurring PTSD and pain is needed. Given our sample is combat veterans only, it is unclear if our results will extend to other trauma populations (such as civilian, natural disasters, sexual assault, or motor vehicle accident). Nuanced information regarding each participant’s pain experience, such as type and number of pain conditions (e.g. primary pain or inflammatory pain condition) or pain chronicity (acute or chronic), was not assessed. Thus, we cannot fully describe the experience of pain for our sample or the impact of treatment on specific pain conditions. Lumping acute and chronic pain may have contributed to overall reduction in pain. Finally, given that all groups showed reduction in pain, observed change may not be attributable to any of the interventions but to some other factor such as regression to the mean, context variables, or meeting with a professional. However, this does provide evidence that additional detailed study of pain in PTSD and PTSD treatment is needed. Investigation into how PTSD interventions address pain across common pain conditions (i.i.e. migraines, lower back pain, fibromyalgia, endometriosis pelvic pain, arthritis) could guide future intervention development. Future research should also investigate whether some interventions work better for acute versus chronic pain or certain pain classifications (nociceptive, neuropathic, nociplastic, etc.). Future research could explore these possibilities.

In conclusion, pain interference and severity showed reduction across PTSD treatments. While it is uncertain if this effect is due to the treatments themselves, it may indicate that PTSD-focused treatments can be associated with a reduction in pain even when they lack a specific pain focus. Additional high-quality studies are needed to further understand the impact of PTSD treatment on pain. Specifically, mediation-focused designs are needed to conclusively determine pathways for PTSD treatment effects on pain, and how these mechanisms may vary with different types of pain sources and characteristics. Relatedly, additional research is needed to determine whether modification or augmentation with pain-specific interventions is needed to maximize response to both PTSD and pain.

## Data Availability

All data are available upon reasonable request to the corresponding author. The participants of this study did not give written consent for their data to be shared publicly, so due to the sensitive nature of the research, supporting data is not available.
